# O.3.2-5 24-hour compositions of physical (in)activity of women with temporomandibular disorders

**DOI:** 10.1093/eurpub/ckad133.150

**Published:** 2023-09-11

**Authors:** Luiz A Brusaca, Luiz Felipe Tavares, Letícia Bojikian Calixtre, Francisco Locks, Ana Beatriz Oliveira

**Affiliations:** Department of Physical Therapy, Federal University of São Carlos, São Carlos, Brazil; Department of Physical Therapy, Federal University of São Carlos, São Carlos, Brazil; Department of Physical Therapy, Federal University of São Carlos, São Carlos, Brazil; Department of Physical Therapy, University of Pernambuco, Petrolina, Brazil; augustobrusaca@gmail.com; Department of Physical Therapy, University of Pernambuco, Petrolina, Brazil; augustobrusaca@gmail.com; Department of Physical Therapy, Federal University of São Carlos, São Carlos, Brazil

## Abstract

**Purpose:**

Physical inactivity is a known risk factor for worsening pain, yet accelerometry-based assessment of sedentary behaviour (SED), physical activity (PA) and time-in-bed (as a proxy for sleep) in individuals with temporomandibular disorders (TMD) are still scarce. We aimed to document and compare the 24-hour time-use compositions of physical behaviours of women with TMD during weekdays and weekends.

**Methods:**

Behaviours were monitored over 7 days using a thigh-worn accelerometer in 44 women with TMD according to the Diagnose Criteria for TMD (mean age 29.6 ± 7.2 years). Daily time-use compositions were exhaustively described in terms of SED in short (<30 min) and long (≥30 min) bouts, light PA (LPA), moderate-to-vigorous PA (MVPA), and time-in-bed. Following a compositional data analysis, isometric log-ratios (ilr) were calculated; ilr_1_ – time-in-bed relative to time spent awake, ilr_2_ – SED (short *and* long bouts) relative to LPA and MVPA, ilr_3_ – SED in short relative to long bouts, and ilr_4_ – LPA relative to MVPA. Differences between weekday and weekend data were examined using repeated-measures MANOVA, followed by univariate post-hoc tests of pairwise differences.

**Results:**

During the week, women with TMD appeared to spend more time SED in short bouts (241 min), more time SED in long bouts (416 min), less time in LPA (241 min), less time in MVPA (62 min), and more time-in-bed (480 min) than on the weekend (223, 379, 276, 70 and 493 min, respectively). The MANOVA showed that the set of ilrs as a whole for weekdays and weekend differed significantly (η_p_^2^ = 0.25, *p* = 0.02). The post-hoc tests showed that time spent SED in short and long bouts relative to LPA and MVPA (ilr_2_) was larger during the week than at the weekend (Cohen’s *d* = 0.31, *p* = 0.04).

**Conclusions:**

Women with TMD behaved differently during weekdays and weekends, whether this relates to days with/without pain or to other factors is still unclear. Further research is required to understand whether these differences in behaviours are part of everyday life or a consequence of musculoskeletal pain.

